# Linkage Disequilibrium Mapping of *CHEK2:* Common Variation and Breast Cancer Risk

**DOI:** 10.1371/journal.pmed.0030168

**Published:** 2006-05-09

**Authors:** Kristjana Einarsdóttir, Keith Humphreys, Carine Bonnard, Juni Palmgren, Mark M Iles, Arvid Sjölander, Yuqing Li, Kee Seng Chia, Edison T Liu, Per Hall, Jianjun Liu, Sara Wedrén

**Affiliations:** **1**Department of Medical Epidemiology and Biostatistics, Karolinska Institutet, Stockholm, Sweden; **2**Population Genetics, Genome Institute of Singapore, Singapore; **3**Department of Mathematics, Stockholm University, Stockholm, Sweden; **4**Genetic Epidemiology Division, Cancer Research UK Clinical Centre, St James's University Hospital, Leeds, United Kingdom; **5**Center for Molecular Epidemiology, Department of Community, Occupational and Family Medicine, National University of Singapore, Singapore; University of MelbourneAustralia

## Abstract

**Background:**

Checkpoint kinase 2 (CHEK2) averts cancer development by promoting cell cycle arrest and activating DNA repair in genetically damaged cells. Previous investigation has established a role for the *CHEK2* gene in breast cancer aetiology, but studies have largely been limited to the rare *1100delC* mutation. Whether common polymorphisms in this gene influence breast cancer risk remains unknown. In this study, we aimed to assess the importance of common *CHEK2* variants on population risk for breast cancer by capturing the majority of diversity in the gene using haplotype tagging single nucleotide polymorphisms (tagSNPs).

**Methods and Findings:**

We analyzed 14 common SNPs spanning 52 kilobases (kb) of the *CHEK2* gene in 92 Swedish women. Coverage evaluation indicated that these typed SNPs would efficiently convey association signal also from untyped SNPs in the same region. Six of the 14 SNPs predicted well both the haplotypic and single SNP variations within *CHEK2*. We genotyped these six tagSNPs in 1,577 postmenopausal breast cancer cases and 1,513 population controls, but found no convincing association between any common *CHEK2* haplotype and breast cancer risk. The *1100delC* mutation was rare in our Swedish population—0.7% in cases and 0.4% in controls—with a corresponding odds ratio for carriers versus noncarriers of 2.26 (95% confidence interval, 0.99–5.15). Estimates of the population frequency and the odds ratio of *1100delC* indicate that our sample is representative of a Northern European population.

**Conclusions:**

Notwithstanding the involvement of the *CHEK2* gene in breast cancer aetiology, we show that common polymorphisms do not influence postmenopausal breast cancer risk.

## Abstract 2

Rare mutations in the *CHEK2* gene are involved in familial breast cancer. This large study among Swedish women did not find an association between common *CHEK2* variants and breast cancer.

## Introduction

Breast cancer is overall the most common cancer in women worldwide [ 1]. Twin and family studies have clearly demonstrated the importance of genetic contribution to breast cancer risk [ 2, 3]. Genetic studies of familial breast cancer have identified several high-penetrance mutations in genes such as *BRCA1* and *BRCA2,* but these mutations contribute little to risk at the population level due to their low prevalence. Whilst genetic risk factors for breast cancer in women not carrying any high-penetrance mutations are largely unknown, a polygenic model has been suggested to account for residual familial risk [ 4], which anticipates small effects of low-penetrance genetic risk variants in combination with environmental influence.

*CHEK2,* encoding a cell-cycle checkpoint kinase, is a strong candidate gene for cancer susceptibility. Following phosphorylation of CHEK2 by ATM after DNA damage, the activated CHEK2 protein phosphorylates p53, Cdc25, and BRCA1, thereby arresting the cell cycle and activating DNA repair [ 5– 8]. As recently verified, the cellular surveillance system for DNA damage-checking and repair plays an important role in averting cancer development [ 9, 10].

An association between the CHEK2* *1100delC* variant and breast cancer risk was initially established in breast cancer families without *BRCA1* or *BRCA2* mutations [ 11, 12]. This deletion, first detected in a breast cancer case with a family history of the Li-Fraumeni syndrome [ 13], leads to a premature termination of translation that abolishes CHEK2 kinase activity [ 14]. Most subsequent population studies of the *1100delC* mutation included few carriers and found no significant association with breast cancer risk [ 12, 15– 23]. Recently, the association between the *1100delC* and breast cancer susceptibility was convincingly replicated in a pooled analysis of 10,860 unselected cases and 9,065 controls from ten studies in five countries [ 24]. In addition, a more recent study has further demonstrated that CHEK2* *1100delC* confers an even higher lifetime risk in women with a family history of bilateral breast cancer [ 25]. Two other putatively functional *CHEK2* single nucleotide polymorphisms (SNPs), *IVS2+1G-A* and *I157T,* have been studied in relation to breast cancer risk. Some groups have found an increased risk of breast cancer associated with these variants [ 15, 23, 26, 27], whilst others have not [ 16, 28, 29]. These polymorphisms are rare and could only make a limited contribution to the genetic risk for breast cancer at the population level. Of the two common *CHEK2* polymorphisms studied so far, neither was associated with breast cancer risk [ 30]. Thus, whether common variants in *CHEK2* are involved in breast cancer aetiology remains unknown.

To address this question, we analyzed the association between common haplotypes in the *CHEK2* gene and breast cancer risk in a well-defined Swedish population consisting of 1,577 breast cancer cases and 1,513 controls. In addition, we explored interactions between *CHEK2* variation and hormone-related risk factors for breast cancer. For comparison with other populations, we also genotyped *1100delC* in the entire set of cases and controls.

## Methods

### Study Population

This study builds on an earlier population-based Swedish case-control study of postmenopausal breast cancer [ 31– 35], with a study base consisting of all Swedish-born women between 50 and 74 years of age that were resident in Sweden between October 1993 and March 1995. During that period, we identified all breast cancer cases at diagnosis through the six regional cancer registries in Sweden, to which reporting of all malignant tumours is mandatory. We randomly selected controls from the Swedish Registry of Total Population that matched the cases in 5-y age strata. Of the eligible cases (3,979) and controls (4,188), 3,345 (84%) cases and 3,454 (82%) controls participated in the initial questionnaire-based study providing detailed information about menopausal hormone use, reproductive history, and other lifestyle factors.

From this initial study, we randomly selected 1,500 cases and 1,500 age-frequency matched controls among the postmenopausal participants without any previous malignancy (except carcinoma in situ of the cervix or nonmelanoma skin cancer). With the intention of increasing statistical power in subgroup analyses, we further selected all remaining cases and controls (191 cases and 108 controls) who had used menopausal hormones (oestrogen only or any combination of oestrogen and progestin) for at least 4 y, and all (110 cases and 104 controls) with self-reported diabetes mellitus. Additionally, 345 controls that were shared between the initial breast cancer study and an endometrial cancer study with same inclusion criteria were added to our control sample. In total, we selected 1,828 cases and 2,057 controls.

Upon providing informed consent, participants donated whole blood. We collected archived paraffin-embedded, non-cancerous tissue samples for deceased breast cancer cases as well as for those breast cancer cases that declined to donate blood but consented to our use of the tissue. We obtained blood samples and archived tissue samples for 1,321 and 275 breast cancer patients, respectively, and blood samples from 1,524 controls. Reasons for nonparticipation included a lack of interest in research or a negative attitude towards genetic research, old age, and severe disease or death. For deceased cases and cases that consented to our access to tissue, the major reason for nonparticipation was unwillingness or lack of time at the respective pathology departments to retrieve the tissue. Population-based participation rates (taking into account the proportion that did not participate in the questionnaire study) for cases and controls were 73% and 61%, respectively.

This study was approved by the Institutional Review Boards in Sweden and at the National University of Singapore.

### DNA Isolation

DNA was extracted by the Swegene laboratories in Malmö (Sweden) from 4 ml of whole blood using the QIAamp DNA Blood Maxi Kit (Qiagen, Valencia, California, United States) according to the manufacturer's instructions. From nonmalignant cells in paraffin-embedded tissue, we extracted DNA using a standard phenol/chloroform/isoamyl alcohol protocol [ 36]. We successfully isolated DNA from 1,318 (blood) and 272 (tissue) breast cancer patients, and from 1,518 controls.

### SNP Markers and Genotyping

We selected SNPs in the *CHEK2* gene and its 5-kb flanking sequences from dbSNP (build 123, http://www.ncbi.nlm.nih.gov/SNP) and Celera databases, aiming for an initial marker density of at least one SNP per 5 kb. SNPs were genotyped using the Sequenom primer extension-based assay (San Diego, California, United States) following the manufacturer's instructions. Briefly, multiplex primer extension assays were designed with the SpectroDesigner software. As template, 5 ng of genomic DNA was used in a multiplex PCR reaction. The PCR product was further purified before the primer extension reaction to generate allele-specific base extension products. The base-extension products were detected in the MassARRAY time-of-flight mass spectrometry (MALDI-TOF) system (Sequenom) to determine genotypes. All genotyping results were generated and checked by laboratory staff unaware of case-control status. Only SNPs for which more than 85% of the samples gave a genotype call were used in further analyses. We genotyped 200 randomly selected SNPs in the 92 control samples using both the Sequenom system and the BeadArray system from Illumina (San Diego, California, United States). The genotype concordance was over 99.5%, suggesting high genotyping accuracy.

### Linkage Disequilibrium Characterization and TagSNP Selection

To characterize the linkage disequilibrium (LD) pattern, we calculated pairwise *D*′ and *R* ^2^ values using two-SNP haplotypes inferred via the EH plus program [ 37]. We reconstructed haplotypes using the PLEM algorithm [ 38] implemented in the *tagSNPs* program [ 39], and selected tagSNPs based on the *R*_h_^2^ coefficient. *R*_h_^2^ is the squared correlation between true haplotype dosage (number of copies of a haplotype) and the haplotype dosage predicted by tagSNPs. We chose tagSNPs so that common SNP genotypes and common haplotypes were predicted with an *R*_h_^2^ value of 0.8 or higher [ 40]. In order to evaluate our tagSNP performance in capturing unobserved SNPs within *CHEK2,* and to assess whether we needed a denser set of markers, we performed the SNP-dropping analysis [ 41]. In brief, each of the 14 genotyped SNPs was dropped in turn, and tagSNPs were selected from the remaining 13 SNPs so that their haplotypes predicted the 13 SNPs with an *R* ^2^ value of 0.9 [ 42]. A slightly more stringent value of 0.9 was used here, as we were predicting only SNPs and not haplotypes with our tagSNPs. We then estimated how well the tagSNP haplotypes of the remaining 13 SNPs predicted the dropped SNP, an evaluation that can provide an unbiased and accurate estimate of tagSNP performance [ 43]. This method assumes the markers to be a random selection of the polymorphisms in the gene. However, some intervals between adjacent genotyped SNPs exceeded 5 kb. We therefore employed additional tests for these intervals. If the tagSNPs selected using only the set of SNPs on one side of the interval predicted the individual SNPs on the other side sufficiently well, then we judged the coverage to be adequate. We had five intervals greater than 5 kb, thus ten sets of predictions. One of these ten was ignored, as there were only two SNPs to the left of the gap.

### Statistical Analyses

We first conducted the haplotype-based association analysis, since the tagSNPs were chosen primarily such that their haplotypes can accurately predict common SNPs in the gene. Our testing strategy was to first fit a single model and to assess for each stratum the haplotype-trait association as a global likelihood ratio test (on 7 degrees of freedom). The global test corresponds to carrying out seven tests on 1 degree of freedom each, and applying a correction for multiple hypothesis testing. Only if the global test was significant did we proceed to testing individual haplotype contrasts, adjusting for multiple testing [ 44]. We first computed expected haplotype dosage using haplotype frequencies estimated for cases and controls combined, assuming Hardy-Weinberg equilibrium (HWE). We then included the haplotype dosage as a covariate in a logistic regression model conditioned on the variables used for sampling: age (5-y age groups), menopausal oestrogen only use (never, < 4 y, or ≥ 4 y), menopausal oestrogen and progestin use (never, < 4 y, or ≥ 4 y) and self-reported diabetes mellitus (yes or no). The appropriateness of the approach, which in imputing haplotypes does not condition on disease status and the nonrandom ascertainment scheme, is argued for by Stram et al. [ 39]. That is, when *R*_h_^2^ values are high, as is the case here, point and interval estimates obtained by this approach will be reasonably accurate. Confounding has been defined as the presence of a common cause to the exposure and the outcome [ 45], and we believe that the lifestyle and reproductive breast cancer risk factors, shown in Table 1, are unlikely to cause genetic variation in *CHEK2*. However, to assess whether the risk factors could be intermediates in the causal pathway between *CHEK2* and breast cancer, we tested whether there was an association between the tagSNPs and any of the risk factors among the randomly sampled controls using a Kruskal-Wallis test. We also assessed the associations between groups of haplotypes and breast cancer risk. Since there is no biological reason to cluster haplotypes on the basis of their frequency, we employed a Bayesian association mapping approach [ 46] that clusters haplotypes according to their allelic similarity.

**Table 1:**
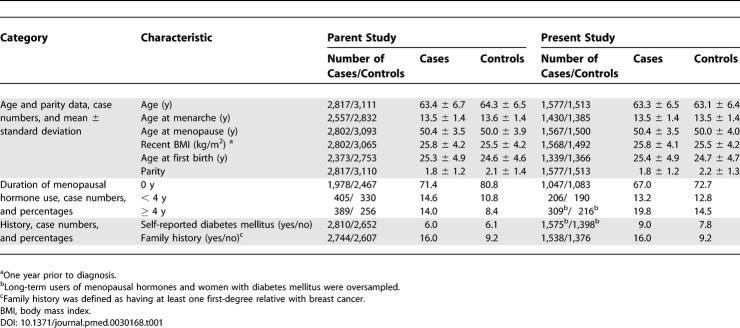
Selected Characteristics of the Postmenopausal Sporadic Breast Cancer Cases and Controls Participating in the Present Study Compared to All Who Answered the Questionnaire in the Parent Study

## Results

### Study Population

Selected characteristics of the participants in the parent questionnaire study and the current genetic study are summarized in Table 1. The distribution of nongenetic factors among cases and controls in the current study were comparable to those in the parent study and reflected established associations [ 47, 48]. The exceptions were long-term menopausal hormone use and diabetes mellitus, which had been consciously oversampled into the current study.

Cases who donated a tissue sample were on average 1.5 y older and were more likely to have been diagnosed with stage 2 or more advanced cancers ( *p* < 0.0001) compared to cases who donated a blood sample. However, no significant differences in genotype frequencies were evident between cases who participated via blood or tissue samples (unpublished data). Furthermore, genotype frequencies were similar between tumours cm or smaller in diameter without lymph node involvement and tumours larger than 2 cm or with spread to regional lymph nodes (unpublished data).

### LD Pattern and Coverage Estimation

**Figure 1:**
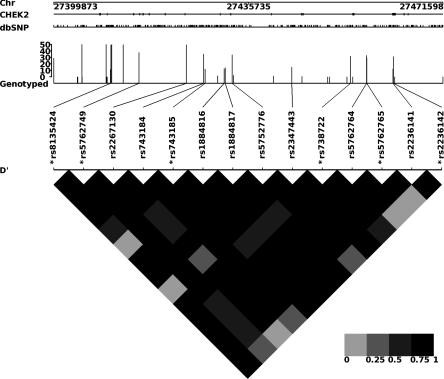
Exon, SNP, and LD Information for *CHEK2* Lane 1 (Chr): Physical position on chromosome 22. Lane 2 (CHEK2): Exon locations. Lane 3 (dbSNP): SNPs downloaded from dbSNP build 124. Lane 4 (Genotyped): SNPs genotyped in our study and their respective frequencies in 92 controls. Accession numbers are given for the 14 common SNPs (MAF ≥ 0.03) that were in HWE, tagSNPs are marked with asterisks. LD grid: pairwise D′ between the 14 common SNPs.

Of 34 successfully genotyped SNPs in *CHEK2,* 23 were polymorphic ( Figure 1 and Table 2), but five of them showed a significant deviation ( *p* < 0.01) from HWE and were therefore excluded from further analyses. The five problematic SNPs were all located within the shared region between *CHEK2* and several homologous pseudogenes [ 49]. Of the remaining 18 polymorphic SNPs, 14 were common (here defined as having a minor allele frequency [MAF] of at least 0.03) and were therefore used to characterize the LD pattern and haplotype structure within *CHEK2*. The 14 SNPs spanned 52 kb of the gene, which yielded a mean and median density of one SNP per 4 kb and 3.2 kb, respectively. The D′ pattern ( Figure 1) suggested strong LD across *CHEK2* without extensive recombination, and we therefore treated the whole gene as a single haplotype block in our subsequent haplotype reconstruction. We identified a total of 31 haplotypes from the 14 SNPs, six of which were common (i.e., had an estimated population frequency of at least 0.03) and accounted for 81% of the chromosomes ( Table 3). We selected six tagSNPs from the 14 common SNPs ( Figure 1 and Table 2) that could efficiently and accurately predict the eight remaining common SNPs and the six common haplotypes within *CHEK2* with an average *R*_h_^2^ of 0.94.

**Table 2:**
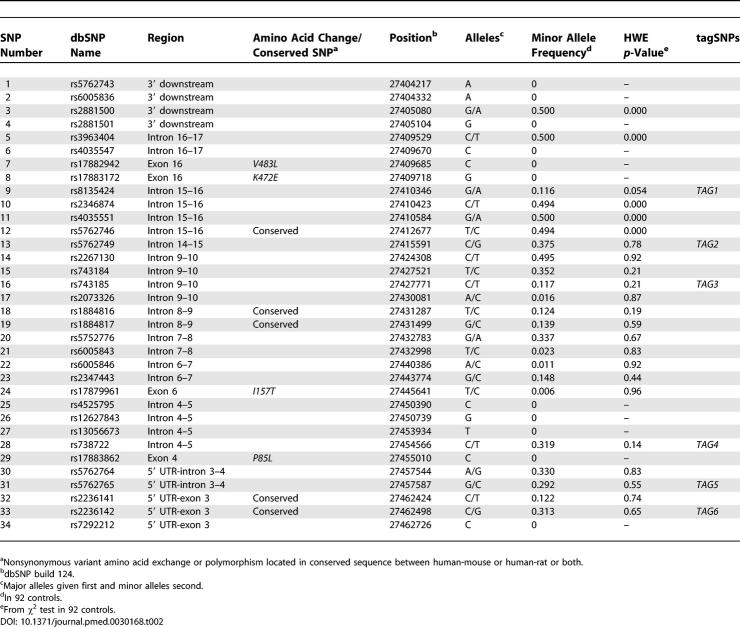
Summary Data on 34 SNPs in *CHEK2* and Its 5-kb Flanking Sequences Successfully Genotyped in 92 Swedish Controls

**Table 3:**
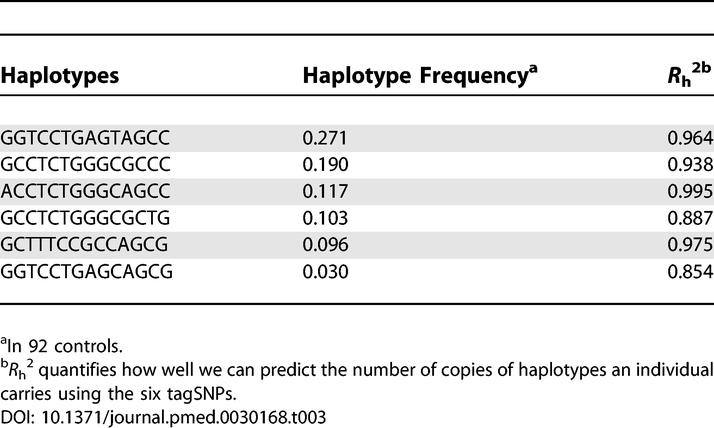
Common Haplotypes Reconstructed Using 14 SNPs in *CHEK2*

Four of the 34 typed *CHEK2* SNPs were nonsynonymous coding polymorphisms ( Table 2). None of these polymorphisms were common and, except for the *1100delC* mutation, were not analyzed further.

Since one cannot assume that tagSNPs capture nongenotyped SNPs as efficiently as genotyped SNPs [ 50], we used the SNP dropping method [ 41] to evaluate tagSNP performance. We found that the dropped SNPs were captured with an average *R* ^2^ of 0.93, with 13 of the 14 dropped SNPs (93%) having an *R* ^2^ greater than 0.75. Therefore, the six selected tagSNPs could efficiently capture not only all the genotyped but also unobserved common SNPs within *CHEK2*. Some intervals between adjacent SNPs exceeded 5 kb. We found the average proportion of the variance on one side of the intervals captured by tagSNPs chosen from the other side was at least 0.68 and averaged 0.75. This is likely to be an underestimate, as the markers considered were a much greater distance apart than the polymorphisms in the intervals of interest. Thus we consider such gaps to present little problem and conclude that our gene is sufficiently densely genotyped and that our tagSNPs effectively capture the common variation present in the gene.

### Association Analyses

We successfully genotyped the six tagSNPs in 1,577 breast cancer cases and 1,513 controls. All six tagSNPs were in HWE among the controls (unpublished data). We assessed, among the randomly selected controls, whether any of the tagSNPs were associated with the lifestyle and reproductive breast cancer risk factors shown in Table 1. *TAG2* and *TAG4* were statistically associated with parity ( *p* = 0.029 and *p* = 0.009, respectively), which showed a statistically significant trend for allelic dose ( *TAG2, p* = 0.006; *TAG4, p* = 0.0002).

We identified 19 rare ( Table S1) and seven common haplotypes from the six tagSNPs ( Table 4). The 19 rare haplotypes were collapsed into a single group in the association analyses. One additional common haplotype was identified in the full set of controls, which was not identified in the preliminary 92 controls. A global test of association, including all common haplotypes and the 19 rare haplotypes as one group in the model, was not statistically significant (likelihood ratio test, *p* = 0.19). Table 4 presents odds ratios (ORs) and confidence intervals (CIs) for tagSNP haplotypes using the most common haplotype (haplotype 1) as reference. None of the common haplotypes increased the risk of breast cancer, but the comparison between the group of 19 rare haplotypes against the reference showed a slight association with breast cancer risk (OR 1.24; 95% CI, 1.02–1.51), which did not, however, carry over to the global test, in which multiple testing has been formally accounted for. After excluding the *1100delC* carriers ( *n* = 28) from the analysis, this association decreased (OR 1.20; 95% CI, 0.98–1.47), whilst the odds ratios for the common haplotypes remained unchanged. Comparing carriers to noncarriers of each haplotype, instead of using haplotype 1 as reference, did not substantially affect the results (unpublished data). Considering only breast cancer cases eventually diagnosed with a second breast cancer ( *n* = 72) similarly did not provide any convincing association ( Table S2). These findings remained unaltered after restricting the analyses to the randomly selected cases and controls or after including other breast cancer risk factors, including parity, as covariates in the logistic regression models.

**Table 4:**
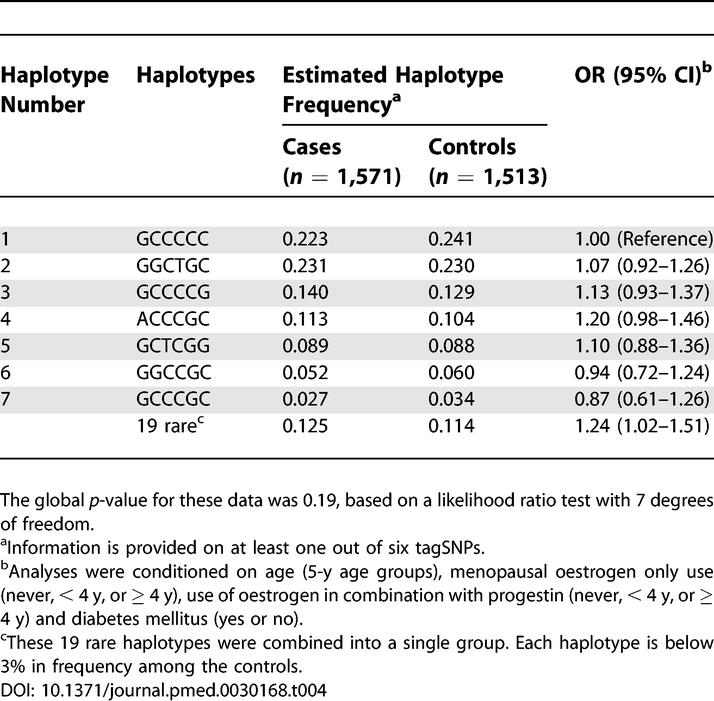
Common Haplotypes Reconstructed Using Six tagSNP in *CHEK2* in Relation to Breast Cancer Risk

We stratified the haplotype analysis by several breast cancer risk factors: first-degree family history, body mass index, age at first birth, menopausal hormone use, and parity ( Tables S3– S7, respectively). None of the stratified analyses yielded a significant (α = 0.05) global test of association.

The 19 rare haplotypes accounted for approximately 20% of the chromosomes. Clustering haplotypes based on their allelic similarity did not provide an indication of a disease susceptibility locus in the region.

We also tested the association between tagSNP genotypes and breast cancer risk, overall ( Table S8) and stratified by other risk factors (unpublished data), but no additional compelling results emerged. Adjusting the models including *TAG2* or *TAG4,* for parity did not change our results.

### Analysis of CHEK2* *1100delC*

The *1100delC* mutation, genotyped in 1,510 cases and 1,334 controls, was rare in our Swedish population, with a frequency of 0.4% in the controls (HWE, *p* = 0.89). The deletion was slightly more common in the cases (0.7%) than in the controls, and the corresponding age- and sampling scheme-adjusted odds ratio for carriers versus noncarriers was 2.26 (95% CI, 0.99–5.15) ( Table 5). The *1100delC* was exclusively carried on rare haplotypes, which may explain the marginally significant association between the group of 19 rare haplotypes and breast cancer risk ( Table 4).

**Table 5:**
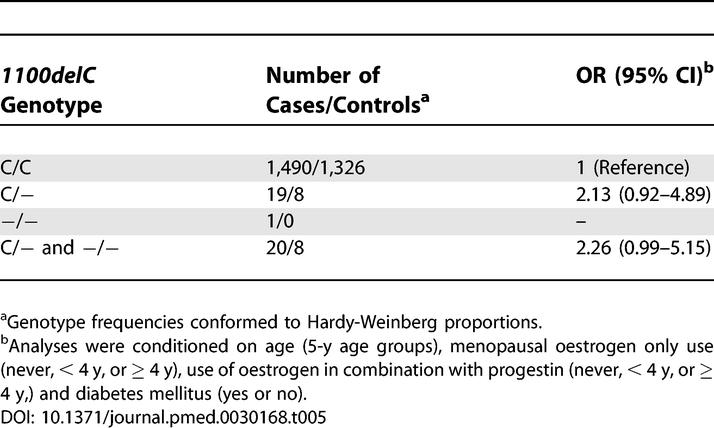
Overall Association of the CHEK2* *1100delC* Mutation with Breast Cancer Risk

## Discussion

Our extensive linkage disequilibrium mapping association study did not provide support for an association between common variations in *CHEK2* and breast cancer risk. The *1100delC* mutation was more common in cases than in controls in our study population, but the difference was not statistically significant. The genotype frequencies were similar to those reported in other populations, thus confirming that our study population is representative of other Northern European populations.

The population on which our study was based is relatively large, well defined, and genetically homogenous. We were able to cover the majority of common variations in the *CHEK2* gene by genotyping a relatively dense map of markers. We had detailed information about hormone-related risk factors for breast cancer that allowed us to explore gene-environment interactions. We also assessed the statistical power of our study. Until now, the power evaluations of *CHEK2* genetic studies have been carried out under the assumption that the genetic risk allele is being directly tested [ 17]. This approach misleadingly overestimates power, because the susceptibility allele is rarely tested directly. Our detailed understanding of the LD pattern across *CHEK2* allowed us to overcome these limitations and estimate power more accurately. To estimate power we used a method described by Chapman et al. [ 42], which assumes codominant effects at an unobserved locus (assuming 80% power, an α-value of 0.05, and *R* ^2^ = 0.9 for the ability of haplotypes to predict the allele count at the causal locus). We estimate that our study should be able to detect a disease susceptibility locus with an odds ratio of 1.23 if its MAF is 0.5, of 1.25 if MAF = 0.35, of 1.32 if MAF = 0.15, or of 1.55 if MAF = 0.05. Therefore, given the comprehensive coverage of common genetic variants within *CHEK2* by our tagSNP approach, it is unlikely that any common disease allele of at least moderate effect was missed in our study.

Selection bias could have arisen in this study, as nonparticipation was related to severe disease or death. However, *CHEK2* genotype frequencies were not significantly different between cases donating tissue samples, representing more ill or deceased patients, and cases donating blood samples. This nonparticipation thus affected only the generalizability of our study.

The *1100delC* variant has not previously been studied in the Swedish population. The variant is rare [ 15– 21, 23], except in a few Northern European populations such as Finland and the Netherlands, where moderate frequency of 1% or above has been observed [ 11, 12, 22]. The low population frequency of *1100delC* in the Swedish population is therefore generally in line with previous data. Furthermore, the corresponding odds ratio for carriers versus noncarriers was 2.26 (95% CI, 0.99–5.15), which is also consistent with the results from other Northern European populations [ 22, 24]. Taken together, the results of our *1100delC* analysis confirm that our case-control sample is representative of a Northern European population.

A few pathogenic variants have been identified by sequencing *CHEK2*, examples of which are *1100delC* and *I157T* [ 13]. Association analyses of these variants in various different populations suggest that these variants are associated with moderate risk for breast cancer and have a low population frequency [ 15, 23, 24, 26, 27]. The pathological effect of *1100delC* was suggested to be due to haploinsufficiency caused by the lost function of the *1100delC* truncated protein [ 51]. In contrast, by introducing a nonconservative amino acid change into the forkhead-associated domain of CHEK2, *I157T* may produce a dominant-negative effect by forming heterodimers with wild-type CHEK2 protein [ 27]. The *I157T* mutation could also have a pathological effect by producing a defective protein for binding and phosphorylating its downstream target, Cdc25A, and for binding p53 and BRCA1 [ 15]. Given the fact that CHEK2 plays a central role in the DNA damage checkpoint and apoptosis pathways [ 52], the modest effect of the *CHEK2* pathogenic variants is surprising. It is however most likely due to the functional redundancy in the crucial DNA damage checkpoint pathway and thus the compensation of defect in CHEK2 function by other members of the pathway. This mechanism was suggested in a recent study showing that the normal degradation of Cdc25A is was shown to be maintained in the carriers of the *1100delC* variant [ 51]. Our association study supports other reports [ 30] indicating that, besides the known pathogenic coding variants, it is unlikely that other common coding or noncoding variants play a significant role in a predisposition to breast cancer. This is consistent with the notion discussed by Cybulski et al. [ 15] that there are three classes of risk genes for breast cancer: a class of genes in which mutations are rare but confer a very high relative risk (including *BRCA1* and *BRCA2*); a class in which variants are common but confer only modest risk (few if any such genes have been confirmed to date); and a class (including *CHEK2*) in which mutations are rare but associated with only modest risk. Although the idea is speculative, *CHEK2* may exercise a pleiotropic effect as a central player in the DNA damage checkpoint and apoptosis pathways on other biological processes than its role in carcinogenesis, and this may provide necessary selection pressure to maintain its functional variants at a low population frequency.

To our knowledge, this is the most comprehensive evaluation of the association between the genetic variation within *CHEK2* and postmenopausal breast cancer risk. However, despite the role of the *CHEK2* gene in breast cancer aetiology, we conclude that common variations in *CHEK2* do not affect breast cancer risk, at least not in the Swedish population. On the other hand, our study cannot exclude the possible influence of rare genetic variants in *CHEK2,* such as the CHEK2* *1100delC,* on breast cancer risk. Investigation of such rare risk alleles in a population-based design requires a much larger sample size than the one utilized in the present study.

## Supporting Information

Alternative Language Abstract S1 Swedish Translation of the Abstract (22 KB DOC)

Alternative Language Abstract S2 Icelandic Translation of the Abstract (21 KB DOC)

Alternative Language Abstract S3 Chinese Translation of the Abstract (27 KB DOC)

Table S1 Association of the 19 Rare Haplotypes with Breast Cancer Risk (49 KB DOC)

Table S2 Common Haplotypes Reconstructed Using Six tagSNPs in *CHEK2* in Relation to Breast Cancer Risk, Considering Only Cases Who Eventually Were Diagnosed with a Second Breast Cancer
(38 KB DOC)

Table S3 Common Haplotypes Reconstructed Using Six tagSNPs in *CHEK2* in Relation to Breast Cancer Risk, by Family History
Family history was considered positive for participants who had at least one first-degree relative with breast cancer.
(45 KB DOC)

Table S4 Common Haplotypes Reconstructed Using Six tagSNPs in *CHEK2* in Relation to Breast Cancer Risk, Stratified by Body Mass IndexBody mass index is calculated as weight/height ^2^ (kg/m ^2^)
(52.5 KB DOC)

Table S5 Common Haplotypes Reconstructed Using Six tagSNPs in *CHEK2* in Relation to Breast Cancer Risk, Stratified by Age at First Childbirth
(51 KB DOC)

Table S6 Common Haplotypes Reconstructed Using Six tagSNPs in *CHEK2* in Relation to Breast Cancer Risk, Stratified by Duration of Use of Any Kind of Medium-Potency Oestrogens
(51 KB DOC)

Table S7 Common Haplotypes Reconstructed Using Six tagSNPs in *CHEK2* in Relation to Breast Cancer Risk, Stratified by Parity
(55 KB DOC)

Table S8 Association between Six tagSNPs in *CHEK2* and Breast Cancer Risk
(47 KB DOC)

### Accession Numbers

The Online Mendelian Inheritance in Man ( http://www.ncbi.nlm.nih.gov/entrez/query.fcgi?db=OMIM) accession numbers for breast cancer is MIM 114480, and for the gene *CHEK2* is MIM 604373.

**Editors' Summary**

**Background.**

Approximately 5% of breast cancer patients have a strong familial risk for the disease. In these families, multiple family members are usually affected, often at an early age (before they are 35). Rare inherited variants in two genes, *BRCA1* and *BRCA2,* are responsible for the high breast cancer risk in most of these “breast cancer families.” Among the other 95% of women with breast cancer who are not known to have inherited one of these high-risk variants, there may still be some inherited susceptibility to the disease: about 10% of women have a “moderate” family history of breast cancer. The inherited genetic factors that are responsible for the more common but weaker susceptibility to breast cancer are so far mostly unknown. These factors could, for example, be common variants in many different genes, each of which on their own cause only a moderately raised risk (although more common variants of the *BRCA1* and *BRCA2* genes do not seem to be associated with breast cancer).

**Why Was This Study Done?**

*CHEK2* is another gene that has been associated with familial breast cancer. In some breast cancer families without mutations in *BRCA1* and *BRCA2,* a specific variant of *CHEK2* (called *1100delC,* indicating a deletion at position 1100) occurred frequently in patients with breast cancer. The association between this particular *CHEK2* variant and an increased risk of breast cancer was confirmed in a large study that looked at approximately 10,000 patients with breast cancer and 10,000 controls. However, the *1100delC* variant of *CHEK2* is rare, and scientists want to know whether more common variants of the gene were associated with breast cancer. A number of previous studies that looked at two common variants of *CHEK2* had not given consistent results. This study used six common variants to predict all common variants of *CHEK2* in a group of postmenopausal Swedish women and tested whether any of the six variants were associated with the women having breast cancer.

**What Did the Researchers Do and Find?**

They identified the six common variants by analyzing the DNA of the *CHEK2* gene in 92 Swedish women. They then compared the frequency of these common variants between approximately 1,500 women with breast cancer and 1,500 women of the same age and similar lifestyles without breast cancer. They found no association between any of the common variants and the likelihood of the women having breast cancer.

**What Do These Findings Mean?**

This study suggests that Swedish women (or those of Northern European descent who have a similar genetic inheritance) who have any of the common variants of the *CHEK2* gene do not have a raised risk of getting breast cancer. However, this finding might not apply in other populations. As in previous studies, this study also found a raised risk of breast cancer for women who carried the rare *1100delC* variant, but the study was too small to test whether there are other rare variants that might also predispose to breast cancer. Overall, scientists have not yet found many common gene variants that increase the risk of getting cancer. But, to test conclusively for associations between genetic variants, both common and rare, and cancer, very large studies will be needed. Understanding how a person's genetic make-up and their environment and lifestyle influence their risk for certain cancers remains a challenge.

**Additional Information.**

Please access these Web sites via the online version of this summary at http://dx.doi.org/10.1371/journal.pmed.0030168.

• Risk factors for breast cancer from the American Cancer Society

• Summary of information on the CHEK2 gene from the US National Center for Biotechnology Information

• Information on Breast Cancer molecular biology and genetics from Cancer Research UK

• Information on breast cancer genetics from the UK Public Health Genetics Unit

## Abbreviations

CHEK2: checkpoint kinase 2
CI: confidence interval
HWE: Hardy-Weinberg equilibrium
kb: kilobase
LD: linkage disequilibrium
MAF: minor allele frequency
OR: odds ratio
SNP: single nucleotide polymorphism
tagSNP: haplotype-tagging single nucleotide polymorphism

## References

[pmed-0030168-b001] Bray F, McCarron P, Parkin DM (2004). The changing global patterns of female breast cancer incidence and mortality. Breast Cancer Res.

[pmed-0030168-b002] Peto J, Mack TM (2000). High constant incidence in twins and other relatives of women with breast cancer. Nat Genet.

[pmed-0030168-b003] Lichtenstein P, Holm NV, Verkasalo PK, Iliadou A, Kaprio J (2000). Environmental and heritable factors in the causation of cancer—Analyses of cohorts of twins from Sweden, Denmark, and Finland. N Engl J Med.

[pmed-0030168-b004] Antoniou AC, Pharoah PD, McMullan G, Day NE, Stratton MR (2002). A comprehensive model for familial breast cancer incorporating BRCA1, BRCA2 and other genes. Br J Cancer.

[pmed-0030168-b005] Matsuoka S, Rotman G, Ogawa A, Shiloh Y, Tamai K (2000). Ataxia telangiectasia-mutated phosphorylates Chk2 in vivo and in vitro. Proc Natl Acad Sci U S A.

[pmed-0030168-b006] Shieh SY, Ahn J, Tamai K, Taya Y, Prives C (2000). The human homologs of checkpoint kinases Chk1 and Cds1 (Chk2) phosphorylate p53 at multiple DNA damage-inducible sites. Genes Dev.

[pmed-0030168-b007] Zeng Y, Forbes KC, Wu Z, Moreno S, Piwnica-Worms H (1998). Replication checkpoint requires phosphorylation of the phosphatase Cdc25 by Cds1 or Chk1. Nature.

[pmed-0030168-b008] Lee JS, Collins KM, Brown AL, Lee CH, Chung JH (2000). hCds1-mediated phosphorylation of BRCA1 regulates the DNA damage response. Nature.

[pmed-0030168-b009] Bartkova J, Horejsi Z, Koed K, Kramer A, Tort F (2005). DNA damage response as a candidate anti-cancer barrier in early human tumorigenesis. Nature.

[pmed-0030168-b010] Gorgoulis VG, Vassiliou LV, Karakaidos P, Zacharatos P, Kotsinas A (2005). Activation of the DNA damage checkpoint and genomic instability in human precancerous lesions. Nature.

[pmed-0030168-b011] Meijers-Heijboer H, van den Ouweland A, Klijn J, Wasielewski M, de Snoo A (2002). Low-penetrance susceptibility to breast cancer due to CHEK2(*)1100delC in noncarriers of BRCA1 or BRCA2 mutations. Nat Genet.

[pmed-0030168-b012] Vahteristo P, Bartkova J, Eerola H, Syrjakoski K, Ojala S (2002). A CHEK2 genetic variant contributing to a substantial fraction of familial breast cancer. Am J Hum Genet.

[pmed-0030168-b013] Bell DW, Varley JM, Szydlo TE, Kang DH, Wahrer DC (1999). Heterozygous germ line hCHK2 mutations in Li-Fraumeni syndrome. Science.

[pmed-0030168-b014] Wu X, Webster SR, Chen J (2001). Characterization of tumor-associated Chk2 mutations. J Biol Chem.

[pmed-0030168-b015] Cybulski C, Gorski B, Huzarski T, Masojc B, Mierzejewski M (2004). CHEK2 is a multiorgan cancer susceptibility gene. Am J Hum Genet.

[pmed-0030168-b016] Friedrichsen DM, Malone KE, Doody DR, Daling JR, Ostrander EA (2004). Frequency of CHEK2 mutations in a population based, case-control study of breast cancer in young women. Breast Cancer Res.

[pmed-0030168-b017] Mateus Pereira LH, Sigurdson AJ, Doody MM, Pineda MA, Alexander BH (2004). CHEK2:1100delC and female breast cancer in the United States. Int J Cancer.

[pmed-0030168-b018] Caligo MA, Agata S, Aceto G, Crucianelli R, Manoukian S (2004). The CHEK2 c.1100delC mutation plays an irrelevant role in breast cancer predisposition in Italy. Hum Mutat.

[pmed-0030168-b019] Osorio A, Rodriguez-Lopez R, Diez O, de la Hoya M, Ignacio Martinez J (2004). The breast cancer low-penetrance allele 1100delC in the CHEK2 gene is not present in Spanish familial breast cancer population. Int J Cancer.

[pmed-0030168-b020] Huzarski T, Cybulski C, Domagala W, Gronwald J, Byrski T (2005). Pathology of breast cancer in women with constitutional CHEK2 mutations. Breast Cancer Res Treat.

[pmed-0030168-b021] Offit K, Pierce H, Kirchhoff T, Kolachana P, Rapaport B (2003). Frequency of CHEK2*1100delC in New York breast cancer cases and controls. BMC Med Genet.

[pmed-0030168-b022] de Jong MM, Nolte IM, Te Meerman GJ, van der Graaf WT, Oosterom E (2005). No increased susceptibility to breast cancer from combined CHEK2 1100delC genotype and the HLA class III region risk factors. Eur J Cancer.

[pmed-0030168-b023] Gorski B, Cybulski C, Huzarski T, Byrski T, Gronwald J (2005). Breast cancer predisposing alleles in Poland. Breast Cancer Res Treat.

[pmed-0030168-b024] CHEK2 Breast Cancer Case-Control Consortium (2004). CHEK2*1100delC and susceptibility to breast cancer: A collaborative analysis involving 10,860 breast cancer cases and 9,065 controls from 10 studies. Am J Hum Genet.

[pmed-0030168-b025] Johnson N, Fletcher O, Naceur-Lombardelli C, dos Santos Silva I, Ashworth A (2005). Interaction between CHEK2*1100delC and other low-penetrance breast-cancer susceptibility genes: A familial study. Lancet.

[pmed-0030168-b026] Bogdanova N, Enbetaen-Dubrowinskaja N, Feshchenko S, Lazjuk GI, Rogov YI (2005). Association of two mutations in the CHEK2 gene with breast cancer. Int J Cancer.

[pmed-0030168-b027] Kilpivaara O, Vahteristo P, Falck J, Syrjakoski K, Eerola H (2004). CHEK2 variant I157T may be associated with increased breast cancer risk. Int J Cancer.

[pmed-0030168-b028] Dufault MR, Betz B, Wappenschmidt B, Hofmann W, Bandick K (2004). Limited relevance of the CHEK2 gene in hereditary breast cancer. Int J Cancer.

[pmed-0030168-b029] Schutte M, Seal S, Barfoot R, Meijers-Heijboer H, Wasielewski M (2003). Variants in CHEK2 other than 1100delC do not make a major contribution to breast cancer susceptibility. Am J Hum Genet.

[pmed-0030168-b030] Kuschel B, Auranen A, Gregory CS, Day NE, Easton DF (2003). Common polymorphisms in checkpoint kinase 2 are not associated with breast cancer risk. Cancer Epidemiol Biomarkers Prev.

[pmed-0030168-b031] Magnusson C, Baron JA, Correia N, Bergstrom R, Adami HO (1999). Breast-cancer risk following long-term oestrogen- and oestrogen-progestin-replacement therapy. Int J Cancer.

[pmed-0030168-b032] Magnusson C, Colditz G, Rosner B, Bergstrom R, Persson I (1998). Association of family history and other risk factors with breast cancer risk (Sweden). Cancer Causes Control.

[pmed-0030168-b033] Magnusson CM, Persson IR, Baron JA, Ekbom A, Bergstrom R (1999). The role of reproductive factors and use of oral contraceptives in the aetiology of breast cancer in women aged 50 to 74 years. Int J Cancer.

[pmed-0030168-b034] Moradi T, Nyren O, Zack M, Magnusson C, Persson I (2000). Breast cancer risk and lifetime leisure-time and occupational physical activity (Sweden). Cancer Causes Control.

[pmed-0030168-b035] Terry P, Wolk A, Persson I, Magnusson C (2001). Brassica vegetables and breast cancer risk. JAMA.

[pmed-0030168-b036] Isola J, DeVries S, Chu L, Ghazvini S, Waldman F (1994). Analysis of changes in DNA sequence copy number by comparative genomic hybridization in archival paraffin-embedded tumor samples. Am J Pathol.

[pmed-0030168-b037] Zhao JH, Curtis D, Sham PC (2000). Model-free analysis and permutation tests for allelic associations. Hum Hered.

[pmed-0030168-b038] Qin ZS, Niu T, Liu JS (2002). Partition-ligation-expectation-maximization algorithm for haplotype inference with single-nucleotide polymorphisms. Am J Hum Genet.

[pmed-0030168-b039] Stram DO, Haiman CA, Hirschhorn JN, Altshuler D, Kolonel LN (2003). Choosing haplotype-tagging SNPS based on unphased genotype data using a preliminary sample of unrelated subjects with an example from the Multiethnic Cohort Study. Hum Hered.

[pmed-0030168-b040] Gabriel SB, Schaffner SF, Nguyen H, Moore JM, Roy J (2002). The structure of haplotype blocks in the human genome. Science.

[pmed-0030168-b041] Weale ME, Depondt C, Macdonald SJ, Smith A, Lai PS (2003). Selection and evaluation of tagging SNPs in the neuronal-sodium-channel gene SCN1A: Implications for linkage-disequilibrium gene mapping. Am J Hum Genet.

[pmed-0030168-b042] Chapman JM, Cooper JD, Todd JA, Clayton DG (2003). Detecting disease associations due to linkage disequilibrium using haplotype tags: A class of tests and the determinants of statistical power. Hum Hered.

[pmed-0030168-b043] Iles MM (2006). Obtaining unbiased estimates of tagging SNP performance. Ann Hum Genet.

[pmed-0030168-b044] Schaid DJ, Rowland CM, Tines DE, Jacobson RM, Poland GA (2002). Score tests for association between traits and haplotypes when linkage phase is ambiguous. Am J Hum Genet.

[pmed-0030168-b045] Hernan MA, Hernandez-Diaz S, Werler MM, Mitchell AA (2002). Causal knowledge as a prerequisite for confounding evaluation: An application to birth defects epidemiology. Am J Epidemiol.

[pmed-0030168-b046] Molitor J, Marjoram P, Thomas D (2003). Fine-scale mapping of disease genes with multiple mutations via spatial clustering techniques. Am J Hum Genet.

[pmed-0030168-b047] Beral V (2003). Breast cancer and hormone-replacement therapy in the Million Women Study. Lancet.

[pmed-0030168-b048] Kelsey JL, Gammon MD, John EM (1993). Reproductive factors and breast cancer. Epidemiol Rev.

[pmed-0030168-b049] Sodha N, Williams R, Mangion J, Bullock SL, Yuille MR (2000). Screening hCHK2 for mutations. Science.

[pmed-0030168-b050] Iles MM (2005). The effect of SNP marker density on the efficacy of haplotype tagging SNPs—a warning. Ann Hum Genet.

[pmed-0030168-b051] Jekimovs CR, Chen X, Arnold J, Gatei M, Richard DJ (2005). Low frequency of CHEK2 1100delC allele in Australian multiple-case breast cancer families: Functional analysis in heterozygous individuals. Br J Cancer.

[pmed-0030168-b052] Ahn J, Urist M, Prives C (2004). The Chk2 protein kinase. DNA Repair (Amst).

