# Supplementary material for: Linkage Disequilibrium Mapping of CHEK2: Common Variation and Breast Cancer Risk
Source: PLoS Med. 2006 May 9;3(6):e168. doi: 10.1371/journal.pmed.0030168 (PMC1457009; doi:10.1371/journal.pmed.0030168)
Supplement: Supplementary file 1 [file pmed.0030168.sd001.doc]

## Kartläggning av vanliga genetiska varianter i CHEK2-gene och deras betydelse for bröstcancerrisk

Checkpointkinas 2 (CHEK2) är ett cellcykelprotein som har stor betydelse for kontrollen av celldelningen. Om CHEK2 inte fungerar finns en teoretisk risk för utveckling av cancer eftersom genetiskt defekta celler inte hindras från att dela sig vidare. Tidigare studier har kunnat visa att vissa ovanliga mutationer i genen för CHEK2 kan bidra till uppkomst av bröstcancer. Det har hittills varit okänt om också vanliga genetiska varianter av CHEK2, singelnukelotidpolymorfier (SNP), kan vara av betydelse. Även om sådana SNP endast förvantas påverka risken litegrand kan de ha stor betydelse ur ett populationsperspektiv, eftersom de är sa vanliga. Vi typade 14 SNP spridda över 52 Kb i CHEK2-genen. Sex av dessa SNP visade sig vara tillräckliga för att kartlägga majoriteten av variationen i den totala uppsättnigen av 14 SNP. Vi analyserade 1577 postmenopausala bröstcancerpatienter och 1513 bröstcancerfria kontroller för dessa 6 SNP men fann inte att någon av dem övertygande påverkade risken för bröstcancer. Den kända mutationen 1100delC var mycket ovanlig i vår svenska population; 0,7% av fallen och 0,4% av kontrollerna bar mutationen vilket medförde ett odds ratio for bröstcancer på 2.26 (95% konfidensintervall 0.99-5.15). Resultaten gällande 1100delC överensstämmer med tidigare publicerade resultat och visar att våra studiedeltagare är representativa för en nordeuropeisk population. Sammanfattningsvis: Trots att CHEK2-genen visats vara av betydelse för bröstcanceruppkomst kan vi inte påvisa att vanliga varianter av genen inverkar på risken för postmenopausal bröstcancerrisk.
