# Supplementary material for: Linkage Disequilibrium Mapping of CHEK2: Common Variation and Breast Cancer Risk
Source: PLoS Med. 2006 May 9;3(6):e168. doi: 10.1371/journal.pmed.0030168 (PMC1457009; doi:10.1371/journal.pmed.0030168)
Supplement: Supplementary file 2 [file pmed.0030168.sd002.doc]

**Kortlaggning Tengslaójafnvægis í *CHEK2*: Algengur Breytileiki og Áhætta á Brjóstakrabbameini**

# Ágrip

Forsaga: Checkpoint kínasi 2 (CHEK2) getur afstýrt þróun krabbameina með því að stuðla að stöðvun frumuhringsins og virkjun DNA viðgerðakerfis í frumum með skemmt erfðaefni. Eldri rannsóknir hafa leitt í ljós að *CHEK2* genið gegnir hlutverki í myndun brjóstakrabbameins en aftur á móti hafa rannsóknirnar aðallega beinst að hinni sjaldgæfu 1100delC stökkbreytingu. Ekki er vitað hvort algengur fjölbreytileiki (polymorphism) í geninu hafi áhrif á brjóstakrabbameinsáhættu þýða. Vid ákvádum að rannsaka áhrif algengra fjölbreytileika *CHEK2* gensins á brjóstakrabbameinsáhættu með því að nota haplótýpumerkjandi SNP (single nucleotide polymorphism = fjölbreytileiki eins núkleótíðs) til að fanga meirihluta fjölbreytileikans í geninu. Aðferðir og Niðurstöður: Við arfgerðargreindum 14 algenga SNP, sem ná yfir 52 kb af *CHEK2* geninu, í 92 sænskum konum. Mat á umfangi fjölbreytileikanna gaf til kynna að með þessum 14 SNP væri hægt að greina tengsl við áhættu á brjóstakrabbameini einnig frá fjölbreytileikum í geninu sem ekki höfðu verið arfgerðargreindir. Sex af þessum 14 fjölbreytileikum gátu staðið fyrir bæði haplótýpubreytileikann og SNP breytileikann í *CHEK2*. Vid arfgerðargreindum þessa 6 fjölbreytileika í 1577 konum sem greindust með brjóstakrabbamein eftir tíðahvörf og 1513 heilbrigðum viðmiðum en fundum engin tengsl á milli algengra haplótýpa í *CHEK2* og áhættu á brjóstakrabbameini. 1100delC stökkbreytingin var sjaldgæf í okkar sænska þýði, 0.7% á meðal sjúklinganna og 0.4% á meðal viðmiðanna. Samsvarandi líkindahlutfall fyrir arfbera samanborið við stofngerðir var 2.26 (95% ÖM 0.99-5.15). Tíðni 1100delC stökkbreytingarinnar og líkindahlutfallið í okkar þýði gefa til kynna að úrtakið er dæmigert fyrir norður evrópuþjóð. Ályktanir: Án þess að draga úr íhlutun *CHEK2* gensins í myndun brjóstakrabbameins, þá sýnum við fram á að algengar breytingar í geninu hafi ekki áhrif á brjóstakrabbameinsáhættu meðal kvenna sem farið hafa í gegnum tíðahvörf.
