# Supplementary material for: Linkage Disequilibrium Mapping of CHEK2: Common Variation and Breast Cancer Risk
Source: PLoS Med. 2006 May 9;3(6):e168. doi: 10.1371/journal.pmed.0030168 (PMC1457009; doi:10.1371/journal.pmed.0030168)
Supplement: Supplementary file 3 [file pmed.0030168.sd003.doc]

摘要：

背景：在遗传损伤的细胞内，周期关卡激酶2 (CHEK2) 可通过诱导细胞周期停滞和激活DNA修复来防止肿瘤发生。 先前的研究已经证实了CHEK2基因在乳腺癌发病中有作用，但是相关的研究大部分局限于罕见的1100delC突变。是否该基因常见的多态性能够影响乳腺癌的患病风险，目前尚不清楚。本研究的目的是通过应用单倍体标记性单核甘酸多态性(tagSNPs)的方法，捕捉该基因内的主要变异，以便评估*CHEK2*常见多态性对人群乳腺癌患病风险的重要性。方法和结果：我们在92个瑞典女性样本中分析了*CHEK2*基因52kb范围内的14个常见SNPs。标记覆盖率评估（Coverage evaluation）显示这些SNPs能有效地传递来自这一区域内其他尚未分析的SNPs的关联信号，并且其中6 SNPs可很有效地预测*CHEK2*内单倍型及单个SNP变异。 我们然后在1577个绝经后的乳腺癌病例及1513个对照人群中分析了这6个tagSNPs的基因型，但未发现任何令人信服的常见*CHEK2*单倍型与乳腺癌患病风险有关联的证据。1100delC在瑞典人群中尚属罕见，只有0.7%的患者和0.4%的对照人群携带，非携带者与携带者的OR值为2.26 (95%可信区间 0.99-5.15). 1100delC的人群基因型分布频率及OR的估计值表明我们的样本具有北欧人群的代表性。结论：尽管*CHEK2* 基因与乳腺癌病因有关，但是我们的结果显示CHEK2的常见多态性对绝经后女性乳腺癌患病风险没有影响。
