# Supplementary material for: Linkage Disequilibrium Mapping of CHEK2: Common Variation and Breast Cancer Risk
Source: PLoS Med. 2006 May 9;3(6):e168. doi: 10.1371/journal.pmed.0030168 (PMC1457009; doi:10.1371/journal.pmed.0030168)
Supplement: Supplementary file 4 [file pmed.0030168.st001.doc]

Table S1. Association of the 19 rare haplotypes with breast cancer risk.

|  | Estimated frequency | |  |
| --- | --- | --- | --- |
| Haplotypes | Cases  (n = 1571^a^) | Controls  (n = 1513^a^) | OR (CI)^b^ |
| GGCCGG | 0.0276 | 0.0243 | 1.13 (0.78-1.65) |
| GCCCGG | 0.0198 | 0.0194 | 1.29 (0.81-2.05) |
| GGCTGG | 0.0083 | 0.0125 | 0.77 (0.39-1.49) |
| GGTCGG | 0.0065 | 0.0103 | 0.71 (0.36-1.42) |
| GCCTGC | 0.0071 | 0.0071 | 0.90 (0.46-1.78) |
| GGCCCG | 0.0076 | 0.0065 | 1.43 (0.66-3.10) |
| GGCTCG | 0.0068 | 0.0039 | 1.77 (0.75-4.17) |
| GGCTCC | 0.0040 | 0.0028 | 1.49 (0.47-4.71) |
| GCTCGC | 0.0038 | 0.0030 | 1.40 (0.48-4.08) |
| GGCCCC | 0.0037 | 0 | --- |
| GGTCGC | 0.0012 | 0.0005 | 2.47 (0.23-26.02) |
| GCCTCC | 0.0007 | 0 | --- |
| GCTTGG | 0 | 0.0008 | --- |
| GGTTGC | 0.0004 | 0 | --- |
| ACTCGG | 0.0217 | 0.0173 | 1.32 (0.89-1.98) |
| ACCCCC | 0.0033 | 0.0016 | 2.52 (0.55-11.46) |
| ACCTGC | 0.0011 | 0.0029 | 0.42 (0.10-1.74) |
| AGTTGG | 0.0004 | 0.0008 | 0.78 (0.05-13.69) |
| AGCCGG | 0.0009 | 0 | --- |
| Total | 0.1249 | 0.1137 |  |

^a^ Information on at least 1 out of 6 tagSNPs

^b^ Analyses were conditioned on age (5 year age-groups), menopausal estrogen only use (never, <4 years or ≥ 4 years), use of estrogen in combination with progestin (never, <4 years or ≥ 4 years) and diabetes mellitus (yes or no). Odds ratios represent carriers of each haplotype compared to non-carriers.
