# Supplementary material for: Linkage Disequilibrium Mapping of CHEK2: Common Variation and Breast Cancer Risk
Source: PLoS Med. 2006 May 9;3(6):e168. doi: 10.1371/journal.pmed.0030168 (PMC1457009; doi:10.1371/journal.pmed.0030168)
Supplement: Supplementary file 5 [file pmed.0030168.st002.doc]

Table S2. Common haplotypes reconstructed using 6 tagSNP in *CHEK2* in relation to breast cancer risk, considering only cases who eventually were diagnosed with a second breast cancer.

|  | Cases (n=71^a^) | |
| --- | --- | --- |
|  | Controls (n=1513^a^) | |
| Haplotypes | OR | CI^b^ |
| 1 | 1 | Ref |
| 2 | 1.20 | 0.67-2.16 |
| 3 | 1.56 | 0.79-3.08 |
| 4 | 2.00 | 1.04-3.85 |
| 5 | 1.44 | 0.65-3.18 |
| 6 | 2.24 | 0.92-5.48 |
| 7 | 0.65 | 0.12-3.53 |
| 19 rare^c^ | 1.31 | 0.66-2.59 |
| Global p-value^d^ | | 0.44 |

^a^ Information on at least 1 out of 6 tagSNPs

^b^ Analyses were conditioned on age (5 year age-groups), menopausal estrogen only use (never, <4 years or ≥ 4 years), use of estrogen in combination with progestin (never, <4 years or ≥ 4 years) and diabetes mellitus (yes or no).

^c^ 19 rare haplotypes combined into a single group. Each haplotype is below 3% in frequency among the controls.

^d^ Likelihood ratio test with 7 degrees of freedom.
