# Supplementary material for: Linkage Disequilibrium Mapping of CHEK2: Common Variation and Breast Cancer Risk
Source: PLoS Med. 2006 May 9;3(6):e168. doi: 10.1371/journal.pmed.0030168 (PMC1457009; doi:10.1371/journal.pmed.0030168)
Supplement: Supplementary file 6 [file pmed.0030168.st003.doc]

Table S3. Common haplotypes reconstructed using 6 tagSNP in *CHEK2* in relation to breast cancer risk by family history defined as having at least one first degree relative with breast cancer.

|  | Family history | | | | |
| --- | --- | --- | --- | --- | --- |
|  | No | |  | Yes | |
|  | Cases (n=1286^a^) | |  | Cases (n=246^a^) | |
|  | Controls (n=1249^a^) | |  | Controls (n=127^a^) | |
| Haplotypes | OR | CI^b^ |  | OR | CI^b^ |
| 1 | 1 | Ref |  | 1 | Ref |
| 2 | 1.12 | 0.95-1.33 |  | 0.84 | 0.50-1.40 |
| 3 | 1.10 | 0.89-1.36 |  | 1.93 | 0.97-3.87 |
| 4 | 1.25 | 1.01-1.55 |  | 1.05 | 0.55-2.00 |
| 5 | 1.17 | 0.92-1.48 |  | 1.00 | 0.51-1.98 |
| 6 | 0.92 | 0.68-1.22 |  | 1.24 | 0.51-3.03 |
| 7 | 0.93 | 0.63-1.38 |  | 0.72 | 0.24-2.23 |
| 19 rare^c^ | 1.29 | 1.05-1.60 |  | 1.07 | 0.60-1.92 |
| Global p-values^d^ | | 0.13 |  |  | 0.44 |

^a^ Information on at least 1 out of 6 tagSNPs

^b^ Analyses were conditioned on age (5 year age-groups), menopausal estrogen only use (never, <4 years or ≥ 4 years), use of estrogen in combination with progestin (never, <4 years or ≥ 4 years) and diabetes mellitus (yes or no).

^c^ 19 rare haplotypes combined into a single group. Each haplotype is below 3% in frequency among the controls.

^d^ Likelihood ratio test with 7 degrees of freedom.
