# Supplementary material for: Linkage Disequilibrium Mapping of CHEK2: Common Variation and Breast Cancer Risk
Source: PLoS Med. 2006 May 9;3(6):e168. doi: 10.1371/journal.pmed.0030168 (PMC1457009; doi:10.1371/journal.pmed.0030168)
Supplement: Supplementary file 7 [file pmed.0030168.st004.doc]

Table S4. Common haplotypes reconstructed using 6 tagSNP in *CHEK2* in relation to breast cancer risk, stratified by body mass index (weight(kg)/(height(m)^2^)).

|  | Body mass index | | | | | | | |
| --- | --- | --- | --- | --- | --- | --- | --- | --- |
|  | <25 | |  | 25 to <28 | |  | ≥28 | |
|  | Cases (n=758^a^) | |  | Cases (n=392^a^) | |  | Cases (n=412^a^) | |
|  | Controls (n=735^a^) | |  | Controls (n=434^a^) | |  | Controls (n=323^a^) | |
| Haplotypes | OR | CI^b^ |  | OR | CI^b^ |  | OR | CI^b^ |
| 1 | 1 | Ref |  | 1 | Ref |  | 1 | Ref |
| 2 | 0.91 | 0.72-1.16 |  | 1.00 | 0.73-1.36 |  | 1.58 | 1.14-2.21 |
| 3 | 0.90 | 0.68-1.19 |  | 1.15 | 0.78-1.71 |  | 1.71 | 1.10-2.64 |
| 4 | 1.04 | 0.79-1.38 |  | 1.29 | 0.86-1.94 |  | 1.60 | 1.05-2.45 |
| 5 | 0.93 | 0.68-1.27 |  | 1.10 | 0.72-1.67 |  | 1.38 | 0.85-2.25 |
| 6 | 0.72 | 0.48-1.08 |  | 0.81 | 0.47-1.40 |  | 1.44 | 0.82-2.52 |
| 7 | 0.86 | 0.50-1.46 |  | 0.79 | 0.40-1.57 |  | 0.90 | 0.40-2.03 |
| 19 rare^c^ | 1.12 | 0.85-1.49 |  | 1.20 | 0.81-1.79 |  | 1.63 | 1.06-2.49 |
| Global p-values^d^ | | 0.60 |  |  | 0.66 |  |  | 0.06 |

^a^ Information on at least 1 out of 6 tagSNPs

^b^ Analyses were conditioned on age (5 year age-groups), menopausal estrogen only use (never, <4 years or ≥ 4 years), use of estrogen in combination with progestin (never, <4 years or ≥ 4 years) and diabetes mellitus (yes or no).

^c^ 19 rare haplotypes combined into a single group. Each haplotype is below 3% in frequency among the controls.

^d^ Likelihood ratio test with 7 degrees of freedom.
