# Supplementary material for: Linkage Disequilibrium Mapping of CHEK2: Common Variation and Breast Cancer Risk
Source: PLoS Med. 2006 May 9;3(6):e168. doi: 10.1371/journal.pmed.0030168 (PMC1457009; doi:10.1371/journal.pmed.0030168)
Supplement: Supplementary file 8 [file pmed.0030168.st005.doc]

Table S5. Common haplotypes reconstructed using 6 tagSNP in *CHEK2* in relation to breast cancer risk, stratified by age at first childbirth.

|  | Age at first childbirth | | | | | | | |
| --- | --- | --- | --- | --- | --- | --- | --- | --- |
|  | ≤ 24 years | |  | 25-29 years | |  | ≥ 30 years | |
|  | Cases (n=635^a^) | |  | Cases (n=439^a^) | |  | Cases (n=260^a^) | |
|  | Controls (n=728^a^) | |  | Controls (n=429^a^) | |  | Controls (n=209^a^) | |
| Haplotypes | OR | CI^b^ |  | OR | CI^b^ |  | OR | CI^b^ |
| 1 | 1 | Ref |  | 1 | Ref |  | 1 | Ref |
| 2 | 1.28 | 1.01-1.64 |  | 0.89 | 0.65-1.20 |  | 1.06 | 0.67-1.68 |
| 3 | 1.26 | 0.94-1.70 |  | 0.94 | 0.65-1.37 |  | 1.25 | 0.73-2.13 |
| 4 | 1.39 | 1.02-1.88 |  | 1.00 | 0.69-1.44 |  | 1.27 | 0.73-2.20 |
| 5 | 1.19 | 0.86-1.65 |  | 1.16 | 0.76-1.76 |  | 0.77 | 0.43-1.38 |
| 6 | 1.23 | 0.81-1.86 |  | 0.70 | 0.41-1.19 |  | 0.58 | 0.27-1.26 |
| 7 | 1.49 | 0.87-2.54 |  | 0.65 | 0.31-1.36 |  | 0.58 | 0.22-1.52 |
| 19 rare^c^ | 1.53 | 1.14-2.06 |  | 0.99 | 0.68-1.42 |  | 0.84 | 0.49-1.43 |
| Global p-values^d^ | | 0.15 |  |  | 0.69 |  |  | 0.34 |

^a^ Information on at least 1 out of 6 tagSNPs

^b^ Analyses were conditioned on age (5 year age-groups), menopausal estrogen only use (never, <4 years or ≥ 4 years), use of estrogen in combination with progestin (never, <4 years or ≥ 4 years) and diabetes mellitus (yes or no).

^c^ 19 rare haplotypes combined into a single group. Each haplotype is below 3% in frequency among the controls.

^d^ Likelihood ratio test with 7 degrees of freedom.
