# Supplementary material for: Linkage Disequilibrium Mapping of CHEK2: Common Variation and Breast Cancer Risk
Source: PLoS Med. 2006 May 9;3(6):e168. doi: 10.1371/journal.pmed.0030168 (PMC1457009; doi:10.1371/journal.pmed.0030168)
Supplement: Supplementary file 9 [file pmed.0030168.st006.doc]

Table S6. Common haplotypes reconstructed using 6 tagSNP in *CHEK2* in relation to breast cancer risk, stratified by duration of use of any kind of medium potency oestrogens.

|  | Use of medium potency oestrogens, duration | | | | | | | |
| --- | --- | --- | --- | --- | --- | --- | --- | --- |
|  | Never | |  | < 4 years | |  | ≥ 4 years | |
|  | Cases (n=1043^a^) | |  | Cases (n=206^a^) | |  | Cases (n=307^a^) | |
|  | Controls (n=1083^a^) | |  | Controls (n=190^a^) | |  | Controls (n=216^a^) | |
| Haplotypes | OR | CI^b^ |  | OR | CI^b^ |  | OR | CI^b^ |
| 1 | 1 | Ref |  | 1 | Ref |  | 1 | Ref |
| 2 | 1.01 | 0.84-1.21 |  | 1.63 | 0.73-3.62 |  | 1.23 | 0.57-2.67 |
| 3 | 1.01 | 0.81-1.26 |  | 2.07 | 0.80-5.35 |  | 1.83 | 0.73-4.56 |
| 4 | 1.13 | 0.90-1.42 |  | 1.19 | 0.48-2.98 |  | 2.06 | 0.76-5.59 |
| 5 | 1.10 | 0.86-1.42 |  | 2.06 | 0.64-6.58 |  | 0.54 | 0.23-1.25 |
| 6 | 0.86 | 0.63-1.19 |  | 1.67 | 0.45-6.23 |  | 1.25 | 0.36-4.34 |
| 7 | 0.82 | 0.54-1.22 |  | 0.52 | 0.07-3.82 |  | 1.13 | 0.18-6.99 |
| 19 rare^c^ | 1.18 | 0.94-1.49 |  | 1.29 | 0.48-3.46 |  | 1.43 | 0.57-3.61 |
| Global p-values^d^ | | 0.47 |  |  | 0.14 |  |  | 0.53 |

^a^ Information on at least 1 out of 6 tagSNPs

^b^ Analyses were conditioned on age (5 year age-groups), menopausal estrogen only use (never, <4 years or ≥ 4 years), use of estrogen in combination with progestin (never, <4 years or ≥ 4 years) and diabetes mellitus (yes or no).

^c^ 19 rare haplotypes combined into a single group. Each haplotype is below 3% in frequency among the controls.

^d^ Likelihood ratio test with 7 degrees of freedom.
