# Supplementary material for: Linkage Disequilibrium Mapping of CHEK2: Common Variation and Breast Cancer Risk
Source: PLoS Med. 2006 May 9;3(6):e168. doi: 10.1371/journal.pmed.0030168 (PMC1457009; doi:10.1371/journal.pmed.0030168)
Supplement: Supplementary file 10 [file pmed.0030168.st007.doc]

Table S7. Common haplotypes reconstructed using 6 tagSNP in *CHEK2* in relation to breast cancer risk, stratified by parity.

|  | Parity | | | | | | | | | | |
| --- | --- | --- | --- | --- | --- | --- | --- | --- | --- | --- | --- |
|  | 0 children | |  | 1 child | |  | 2 children | |  | ≥3 children | |
|  | Cases (n=233^a^) | |  | Cases (n=340^a^) | |  | Cases (n=601^a^) | |  | Cases (n=397^a^) | |
|  | Controls (n=147^a^) | |  | Controls (n=269^a^) | |  | Controls (n=574^a^) | |  | Controls (n=523^a^) | |
| Haplotypes | OR | CI^b^ |  | OR | CI^b^ |  | OR | CI^b^ |  | OR | CI^b^ |
| 1 | 1 | Ref |  | 1 | Ref |  | 1 | Ref |  | 1 | Ref |
| 2 | 0.72 | 0.44-1.18 |  | 0.93 | 0.64-1.35 |  | 1.00 | 0.77-1.29 |  | 1.45 | 1.06-1.97 |
| 3 | 0.96 | 0.51-1.80 |  | 1.11 | 0.70-1.74 |  | 1.01 | 0.74-1.39 |  | 1.43 | 0.99-2.05 |
| 4 | 1.23 | 0.63-2.41 |  | 0.96 | 0.60-1.53 |  | 1.13 | 0.82-1.56 |  | 1.68 | 1.16-2.44 |
| 5 | 1.12 | 0.53-2.36 |  | 0.97 | 0.58-1.62 |  | 1.08 | 0.77-1.51 |  | 1.46 | 0.96-2.22 |
| 6 | 0.96 | 0.44-2.09 |  | 0.84 | 0.42-1.68 |  | 0.84 | 0.53-1.33 |  | 1.28 | 0.78-2.09 |
| 7 | 0.34 | 0.10-1.13 |  | 0.77 | 0.33-1.81 |  | 1.04 | 0.57-1.89 |  | 1.16 | 0.60-2.23 |
| 19 rare^c^ | 1.55 | 0.81-2.94 |  | 1.10 | 0.71-1.69 |  | 1.15 | 0.83-1.59 |  | 1.16 | 0.60-2.23 |
| Global p-values^d^ | | 0.24 |  |  | 0.98 |  |  | 0.94 |  |  | 0.13 |

^a^ Information on at least 1 out of 6 tagSNPs

^b^ Analyses were conditioned on age (5 year age-groups), menopausal estrogen only use (never, <4 years or ≥ 4 years), use of estrogen in combination with progestin (never, <4 years or ≥ 4 years) and diabetes mellitus (yes or no).

^c^ 19 rare haplotypes combined into a single group. Each haplotype is below 3% in frequency among the controls.

^d^ Likelihood ratio test with 7 degrees of freedom.
