# Supplementary material for: Linkage Disequilibrium Mapping of CHEK2: Common Variation and Breast Cancer Risk
Source: PLoS Med. 2006 May 9;3(6):e168. doi: 10.1371/journal.pmed.0030168 (PMC1457009; doi:10.1371/journal.pmed.0030168)
Supplement: Supplementary file 11 [file pmed.0030168.st008.doc]

Table S8. Association between 6 tagSNPs in *CHEK2* and breast cancer risk.

| tagSNPs | Number of  cases^a^/controls^a^ | OR (CI) ^b^ |
| --- | --- | --- |
| TAG1 (rs8135424) |  |  |
| GG | 1109/1013 | 1 (reference) |
| GA | 344/284 | 1.09 (0.91-1.31) |
| AA | 40/22 | 1.68 (0.99-2.86) |
| TAG2 (rs5762749) |  |  |
| CC | 613/559 | 1 (reference) |
| CG | 684/596 | 1.04 (0.88-1.22) |
| GG | 174/163 | 0.97 (0.76-1.24) |
| TAG3 (rs743185) |  |  |
| CC | 1146/1028 | 1 (reference) |
| CT | 334/285 | 1.06 (0.88-1.27) |
| TT | 21/18 | 1.07 (0.56-2.03) |
| TAG4 (rs738722) |  |  |
| CC | 800/710 | 1 (reference) |
| CT | 562/506 | 0.98 (0.84-1.15) |
| TT | 94/80 | 1.05 (0.76-1.44) |
| TAG5 (rs5762765) |  |  |
| GG | 544/472 | 1 (reference) |
| GC | 709/649 | 0.95 (0.81-1.12) |
| CC | 212/184 | 0.99 (0.78-1.26) |
| TAG6 (rs2236142) |  |  |
| CC | 685/623 | 1 (reference) |
| CG | 637/563 | 1.04 (0.88-1.22) |
| GG | 173/126 | 1.24 (0.96-1.60) |

^a^ Information on at least 1 out of 6 tagSNPs

^b^ Analyses were conditioned on age (5 year age-groups), menopausal estrogen only use (never, <4 years or ≥ 4 years), use of estrogen in combination with progestin (never, <4 years or ≥ 4 years) and diabetes mellitus (yes or no).
